# A Change Of Heart

**DOI:** 10.1007/s12471-014-0566-4

**Published:** 2014-07-09

**Authors:** F. M. Zimmermann, E. van Mierlo, A. Meijer, L. R. Dekker

**Affiliations:** Department of Cardiology, Catharina Hospital Eindhoven, Michelangelolaan 2, 5623 EJ Eindhoven, the Netherlands

## Question

**Fig. 1:**
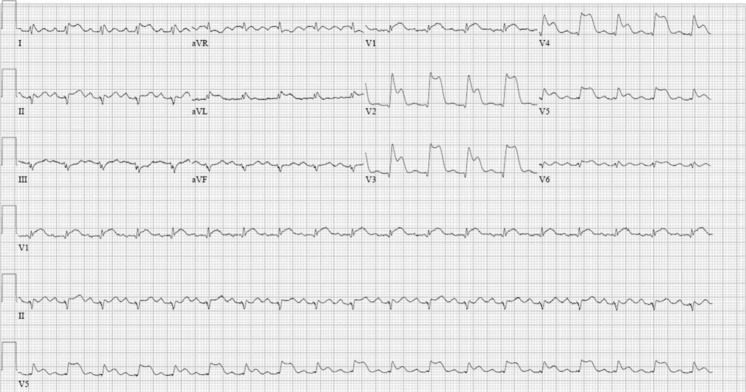
ECG when the chest pain reoccurred

A 52-year-old man, without a cardiac medical history, was admitted to the emergency room with chest pain. Physical examination revealed no abnormalities. On presentation, his chest pain had spontaneously disappeared and the ECG did not show significant abnormalities. Thirty minutes later the chest pain reoccurred. A second ECG at that moment is shown in Fig. 1.

What is your diagnosis and can you explain the mechanism?

You will find the answer elsewhere in this issue.

